# Targeting hydroxycinnamoyl CoA: shikimate hydroxycinnamoyl transferase for lignin modification in *Brachypodium distachyon*

**DOI:** 10.1186/s13068-021-01905-1

**Published:** 2021-02-27

**Authors:** Juan Carlos Serrani-Yarce, Luis Escamilla-Trevino, Jaime Barros, Lina Gallego-Giraldo, Yunqiao Pu, Art Ragauskas, Richard A. Dixon

**Affiliations:** 1 BioDiscovery Institute and Department of Biological Sciences, University of North Texas, Denton, 76203 TX USA; 2 BioEnergy Science Center (BESC), Oak Ridge National Laboratory, Oak Ridge, TN USA; 3 Center for Bioenergy Innovation (CBI), Oak Ridge National Laboratory, Oak Ridge, TN USA; 4 UT-ORNL Joint Institute for Biological Sciences, Oak Ridge, TN USA

**Keywords:** Lignin modification, Phenylpropanoid biosynthesis, Saccharification efficiency, RNA interference, Monocot, NMR analysis

## Abstract

**Background:**

Hydroxycinnamoyl CoA: shikimate hydroxycinnamoyl transferase (HCT) is a central enzyme of the so-called “esters” pathway to monolignols. As originally envisioned, HCT functions twice in this pathway, to form coumaroyl shikimate and then, in the “reverse” direction, to convert caffeoyl shikimate to caffeoyl CoA. The discovery of a caffeoyl shikimate esterase (CSE) that forms caffeic acid directly from caffeoyl shikimate calls into question the need for the reverse HCT reaction in lignin biosynthesis. Loss of function of HCT gives severe growth phenotypes in several dicot plants, but less so in some monocots, questioning whether this enzyme, and therefore the shikimate shunt, plays the same role in both monocots and dicots. The model grass *Brachypodium distachyon* has two *HCT* genes, but lacks a classical *CSE* gene. This study was therefore conducted to evaluate the utility of HCT as a target for lignin modification in a species with an “incomplete” shikimate shunt.

**Results:**

The kinetic properties of recombinant *B. distachyon* HCTs were compared with those from *Arabidopsis thaliana*, *Medicago truncatula*, and *Panicum virgatum* (switchgrass) for both the forward and reverse reactions. Along with two *M. truncatula* HCTs, *B. distachyon* HCT2 had the least kinetically unfavorable reverse HCT reaction, and this enzyme is induced when HCT1 is down-regulated. Down regulation of *B. distachyon* HCT1, or co-down-regulation of HCT1 and HCT2, by RNA interference led to reduced lignin levels, with only modest changes in lignin composition and molecular weight.

**Conclusions:**

Down-regulation of HCT1, or co-down-regulation of both *HCT* genes, in *B. distachyon* results in less extensive changes in lignin content/composition and cell wall structure than observed following HCT down-regulation in dicots, with little negative impact on biomass yield. Nevertheless, HCT down-regulation leads to significant improvements in biomass saccharification efficiency, making this gene a preferred target for biotechnological improvement of grasses for bioprocessing.

## Background

**Fig. 1:**
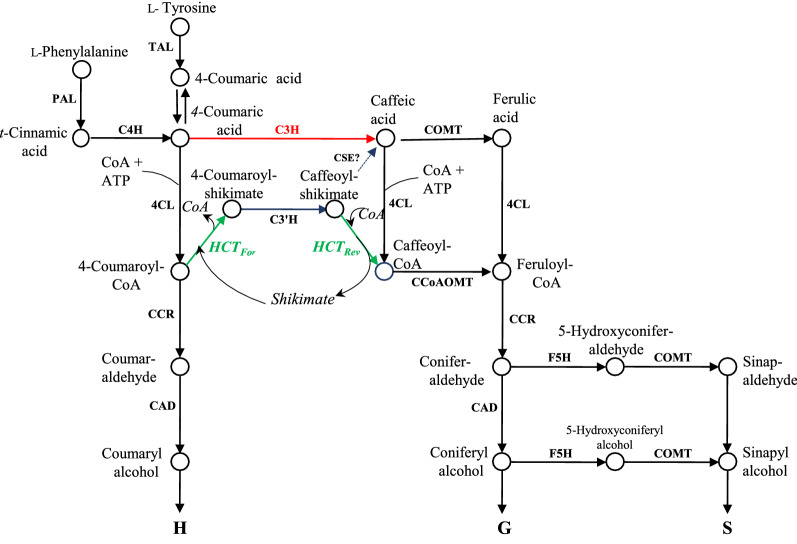
The monolignol pathway in *B. distachyon*. In this model, two different pools of 4-coumarate are shown, one originating from the PAL + C4H reactions, the other directly from the reaction catalyzed by TAL. The two pools are shown as being in equilibrium, but they may not be equivalent [20]. The reactions of the shikimate shunt involving the forward “*HCT*_*For*_” and reverse “*HCT*_*Rev*_” HCT reactions are shown in green. The direct pathway through the non-esterified hydroxycinnamic acids, involving a soluble 4-coumarate 3-hydroxylase [7] is shown in red. The enzymes shown in bold capitals are: PAL, L-phenylalanine ammonia-lyase; TAL, L-tyrosine ammonia-lyase; C4H, cinnamate 4-hydroxylase; HCT, hydroxycinnamoyl CoA: shikimate/quinate hydroxycinnamoyl transferase; 4CL, 4-coumarate:CoA ligase; C3´H, 4-coumaroyl shikimate 3´-hydroxylase (cytochrome P450); C3H, 4-coumarate 3-hydroxylase (ascorbate peroxidase); F5H, ferulate/coniferaldehyde 5-hydroxylase; CCoAOMT, caffeoyl-CoA 3-*O*-methyltransferase; COMT, caffeic acid/5-hydroxyconiferaldehyde 3-*O*-methyltransferase; CCR, cinnamoyl-CoA reductase; CAD, cinnamyl alcohol dehydrogenase. CSE, caffeoyl shikimate esterase [5], reported to be absent in some grass species [6], is shown with a question mark

The pathway to monolignols, the building blocks of the cell wall polymer lignin, has been revised a number of times over the years [1, 2]. Because of the apparent promiscuity of several of the enzymes involved, early models of the pathway presented it as a metabolic grid, in which hydroxylation and/or *O*-methylation of the aromatic ring could take place at different levels of oxidation of the terminal group on the side chain, including on free coumaric and caffeic acids [1]. However, this view changed with the demonstration that the two key enzymes ferulate 5-hydroxylase (F5H) and caffeic acid 3-*O*-methyltransferase (COMT) showed strong kinetic preferences for coniferaldehyde and 5-hydroxyconiferaldehyde, respectively, functions supported by genetic evidence in *A. thaliana* [2] (Fig. 1). It was then shown that the 3-hydroxylation of the aromatic ring could be catalyzed by the coupled activities of a hydroxycinnamoyl CoA: shikimate hydroxycinnamoyl transferase (HCT) and a coumaroyl shikimate 3′-hydroxylase (C3′H), findings that were again supported by genetic evidence [3, 4]. Together, these revisions to the pathway removed caffeic and ferulic acids as lignin pathway intermediates. In this new model caffeoyl CoA, the presumed substrate for introduction of the 3-*O*-methyl group, was proposed to be formed by HCT functioning a second time in the reverse direction to form a CoA ester from a shikimate ester (Fig. 1).

This picture was complicated by the demonstration that a caffeoyl shikimate esterase (CSE) functioned in monolignol biosynthesis in *A. thaliana* [5]. This enzyme generated free caffeic acid, reinstating this molecule as an intermediate in the phenylpropanoid pathway (Fig. 1). Loss of function of CSE in *A. thaliana* results in a strong increase in hydroxyphenyl (H) units in lignin, with overall lignin levels reduced by up to 36% [5]. The corresponding phenotype for loss of function of CSE in the model legume *M. truncatula* is even more severe, suggesting that CSE is essential for monolignol biosynthesis in this species [6]. However, it appears that not all plants possess *CSE* genes or associated enzymatic activity, including some monocots such as rice and the model grass *B. distachyon* [6]. Furthermore, adding CSE to the “esters” pathway in the biosynthesis of monolignols results in the consumption of an extra molecule of ATP for conversion of coumarate to caffeoyl CoA (Fig. 1), making the overall process energetically less favorable than operation of the reverse HCT reaction, which involves a transesterification. The picture has become yet more complicated by the recent demonstration that caffeic acid can be formed through the direct action of a soluble coumarate 3-hydroxylase (C3H) in *B. distachyon* [7], a route that potentially by-passes HCT, C3´H and CSE for monolignol biosynthesis.

Whereas down-regulation of HCT in dicot species results in severe phenotypes with stunted growth and a massive increase in the proportion of H units in lignin [4, 8, 9], emerging data suggest that HCT, and by extension the esters pathway, may not be essential for monolignol biosynthesis in grasses. Thus, although RNAi-mediated down-regulation of late lignin pathway enzymes (COMT, CCR, CAD) resulted in the predicted lignin phenotypes in switchgrass (*Panicum virgatum*), down-regulation of caffeoyl CoA 3-*O*-methyltransferase (CCoAOMT) had little effect on lignin content and composition, and transcript expression data questioned the functions of HCT and C3´H in lignin biosynthesis [10]. It has recently been shown that targeting both *HCT* genes in switchgrass for RNAi-mediated down-regulation results in a less severe lignin and growth phenotype than observed from HCT down-regulation in dicots [11].

We here re-evaluate the importance of the forward and reverse HCT reactions in monolignol biosynthesis, and address the impacts of down-regulating HCT in *B. distachyon*. Our data indicate that the reverse HCT reaction is kinetically unfavorable in both dicots and monocots, and that, in spite of the lack of a specific CSE enzyme, strong down-regulation of HCT in *B. distachyon* results in relatively small effects on lignin content and composition. Nevertheless, HCT appears to be an effective target for improving cell wall saccharification efficiency in *B. distachyon*, without the severely deleterious growth phenotypes observed following down-regulation of this enzyme in dicots.

## Results

### Evaluation of the reverse HCT reaction

We first compared the forward and reverse HCT reactions in crude protein extracts from stems of the dicots *A. thaliana* and *M. truncatula*, and the monocots *P. virgatum* and *B. distachyon*, the latter of which lacks a classical *CSE* gene [6]. The data (Additional file 1: Fig. S1a) confirmed the forward reaction (coumaroyl CoA to coumaroyl shikimate) in extracts from all four species, but the reverse reaction (caffeoyl shikimate to caffeoyl CoA) was either low (*P. virgatum*, *M. truncatula*) or close to undetectable (*B. distachyon*, *A. thaliana*).

**Fig. 2:**
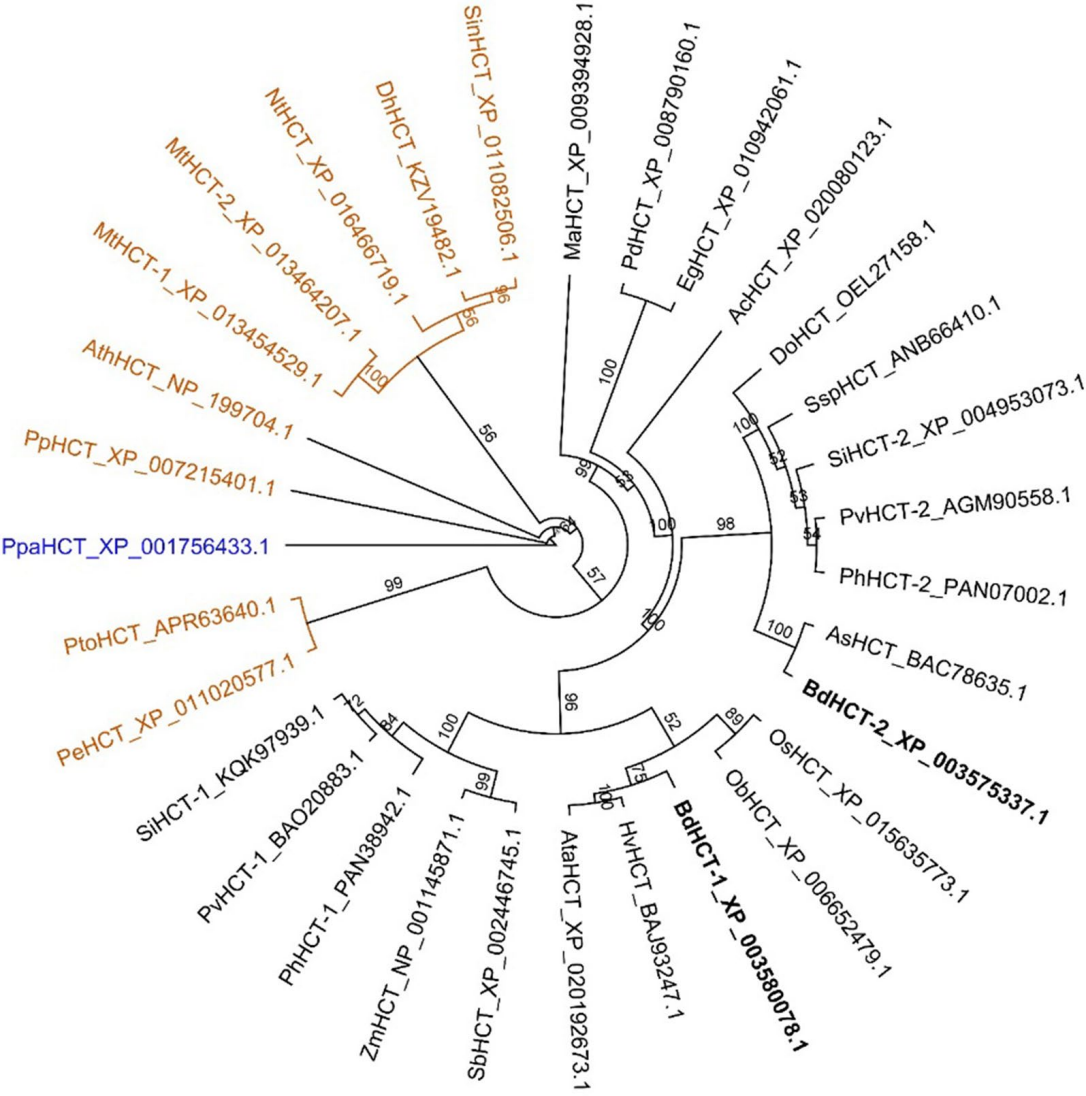
Phylogenetic tree of HCT enzymes in plants. Protein sequences were obtained from GenBank, with the accession numbers shown. Species are: *Aegilops tauschii* (Ata), *Ananas comosus* (Ac), *Arabidopsis thaliana* (Ath), *Avena sativa* (As), *Bambusa emeiensis* (Be), *Brachypodium distachyon* (Bd), *Capsicum annuum* (Ca), *Cynara cardunculus* (Cc), *Dichanthelium oligosanthes* (Do), *Dorcoceras hygrometricum* (Dh), *Dryopteris fragrans* (Df), *Elaeis guineensis* (Eg), *Eucalyptus globulus* (Egl), *Eucalyptus grandis* (Egr), *Festuca rubra* (Fr), *Glycine max* (Gm), *Hordeum vulgare* (Hv), *Lactuca sativa* (Ls), *Medicago truncatula* (Mt), *Musa acuminata* (Ma), *Nicotiana attenuata* (Na), *Nicotiana sylvestris* (Ns), *Nicotiana tabacum* (Nt), *Nicotiana tomentosiformis* (Nto), *Oryza sativa* (Os), *Oryza brachyantha* (Ob), *Panicum hallii* (Ph), *Panicum virgatum* (Pv), *Phoenix dactylifera* (Pd), *Physcomitrella patens* (Ppa), *Populus euphratica* (Pe), *Populus tomentosa* (Pto), *Populus trichocarpa* (Ptr), *Prunus persica* (Pp), *Saccharum spontaneum* (Ssp), *Sesamum indicum* (Sin), *Setaria itálica* (Si), *Solanum tuberosum* (St), *Sorghum bicolor* (Sb). *Spinacia oleracea* (So), *Triticum aestivum* (Ta), *Triticum urartu* (Tu), *Zea mays* (Zm). Monocots are shown in black, dicots in burnt orange and the outgroup used to root the tree in blue. The *B. distachyon* HCT enzymes targeted for genetic studies are shown in bold (BdHCT-1, Bradi5g14720; BdHCT-2, Bradi3g48530). The phylogenetic tree was built by PHYML plugin [39] using JTT substitution model and 1000 bootstraps replicates. Bootstrap support values are indicated at the nodes

Phylogenetic analysis has indicated that *A. thaliana* possesses a single *HCT* gene, whereas *M. truncatula*, *P. virgatum* and *B. distachyon* each possess two *HCT* genes (Fig. 2). The two *B. distachyon HCT* genes are 65 and 62% identical at the amino acid sequence level to the *A. thaliana* HCT. A more detailed phylogenetic analysis of the BAHD family of plant acyltransferases shows that *B. distachyon* also contains a number of HCT-like genes, with less than 40% amino acid identity to *A. thaliana* HCT (Additional file 1: Figs. S2a, b). In spite of this low sequence identity, the basic catalytic His153, Arg356 handle, Thr369, and Trp371 [12] are maintained in some of these HCT-like enzymes (Additional file 1: Fig. S2c). We cloned the open reading frames of the seven HCTs from the four species above into pDEST17 vector for expression in *E. coli* as 6xHis-tagged proteins. After purification by nickel affinity chromatography (Additional file 1: Fig. S1b), the proteins were assayed at a range of substrate concentrations to determine the kinetics for the forward and reverse reactions (Table 1). Because it was difficult to remove contaminating proteins and protein purity had to be estimated from gel scanning and image analysis, Kcat values are given as apparent values. K_M_ values for the forward reactions varied from 5.1 µM (PvHCT2) to 55.2 µM (BdHCT2), whereas K_m_ values for the reverse reactions were much higher, in the range of 56.7 (PvHCT2) to 511.8 (BdHCT1). Based on Kcat_app_/Km values, the least efficient reverse activities were seen for *A. thaliana* HCT and *B. distachyon* HCT1, and the most efficient were *B. distachyon* HCT2 and the two *M. truncatula* HCTs (Table 1).

**Table 1:** Kinetics of recombinant HCTs in the forward and reverse directions

Enzyme	Km (µM^−1^)	Kcat (s^−1^)^a^	Kcat/Km (s^−1^ µM^−1^)
HCT forward: 4-coumaroyl CoA (varying substrate) + shikimic acid (saturating substrate)			
AtHCT1	18.5	17.5	0.95
BdHCT1	32.2	25.3	0.79
BdHCT2	55.2	20.5	0.37
MtHCT1	40.6	40.8	1.01
MtHCT2	44.3	48.2	1.01
*PvHCT1	19.1	60.6	3.13
*PvHCT2	5.1	10.3	2.00
HCT reverse: caffeoyl shikimate (varying substrate) + CoA (saturating substrate)			
AtHCT1	225.0	17.0	0.076
BdHCT1	511.8	41.9	0.082
BdHCT2	63.4	12.0	0.189
MtHCT1	147.7	25.9	0.175
MtHCT2	139.3	31.1	0.223
*PvHCT1	60.6	7.5	0.123
*PvHCT2	56.7	5.2	0.090

*Data taken from [27] Escamilla-Treviño et al., 2014

^a^Kcat values are apparent values in view of the need to estimate purity of enzyme preparations by gel scanning and image analysis

### Tissue-specific lignification and expression of HCT in B. distachyon

Under our greenhouse growth conditions, at 45 days after germination (dag) the *B. distachyon* stem has an average of 8 internodes (numbered from first formed to latest formed, counting up from the crown) that show different patterns of lignin deposition. A central segment from each of the first eight internodes of wild-type plants was isolated for phloroglucinol staining, which reveals total lignin through reaction with cinnamaldehyde end groups [13]. Internodes 3 and 4 showed the strongest staining, whereas the youngest internode (#8) stained less (Additional file 1: Figure S3a). Lignin composition in internodes 5 and 8 was analyzed by thioacidolysis. Total thioacidolysis yield (S + G + H units) was higher in internode 5 than in internode 8, as expected for the more mature internode (Additional file 1: Table S1). On the basis of the above data, we selected internodes 3 to 5 as informative for analysis of subsequent transgenic plants modified in expression of HCT, as they contained a gradient of strongly lignified tissues. It was necessary to pool more than one internode, as tissue was limiting for chemical analysis, particularly in T0 progeny lines.

Interrogation of the Brachypodium gene expression atlas [14], which reports data based on microarray analysis, showed that *HCT1* is most strongly expressed in stem tissue, whereas HCT2 has stronger expression in leaf (Additional file 1: Figure S3b). Roots, leaves, stems and seeds were collected from plants of *B. distachyon* at 15 and 45 dag for analysis of HCT transcript levels by qRT-PCR. The data (Additional file 1: Figure S3c) confirmed that *HCT1* was more highly expressed in mature stems, in which *HCT2* was only weakly expressed. However, our qPCR data indicated that *HCT2* was expressed at a level close to that of *HCT1* in young leaves, but was hardly expressed at all in mature leaves (Additional file 1: Figure S3c), in contradiction to the data in the gene expression atlas (Additional file 1: Figure S3b). Variability in HCT2 transcript levels is discussed further below.

### Generation of *B. distachyon* RNAi lines with reduced expression of HCT

**Fig. 3:**
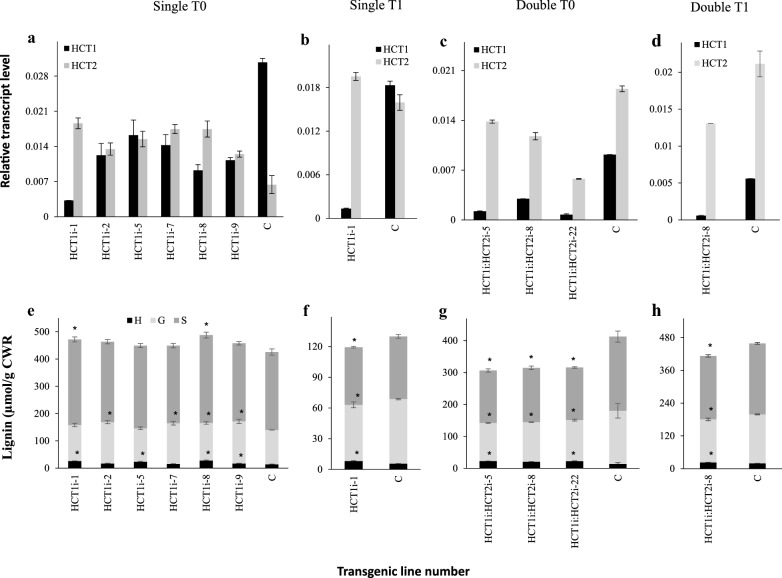
HCT transcript levels and lignin content and composition in stems of *B. distachyon* HCT-RNAi lines. (**a**–**d**) HCT transcript levels relative to housekeeping gene tubulin alpha-1 chain (Bradi1g10150) in T0 (**a**) and T1 (**b**) generation from single HCT1-RNAi lines, and T0 (**c**) and T1 (**d**) generation from double HCT1:HCT2-RNAi lines. **e**–**h** Lignin monomer composition determined by thioacidolysis in lower stem internodes of the same lines as shown in **a**–**d**. H, G, S = yields of the thioethylated products of *p*-hydroxyphenyl (H), guaiacyl (G), and syringyl (S) lignin units expressed as μmol per gram of cell wall residue (CWR). In panels **a**-**d**, all values shown for the HCT-RNAi lines are significantly different (*P* < 0.05) from the wild-type control (WT) plants. For panels **e**–**h**, statistically significant differences (*P* < 0.05) are shown with an asterisk

A 260-bp sequence of HCT1 (Additional file 1: Figure S4a) was selected to generate an RNA silencing construct, which was cloned into the pANIC8A vector (Additional file 1: Figure S4b) and introduced into *B. distachyon* by Agrobacterium-mediated transformation [15]. All nine HCT1-RNAi T0 lines generated exhibited an approximately 50–90% reduction in levels of *BdHCT1* transcript but, surprisingly, levels of *BdHCT2* transcripts were increased (Additional file 1: Figure S5a); six of these T0 lines were selected for further study (Fig. 3a). On the basis of its low HCT1 transcript levels (down-regulated by around 92%) (Additional file 1: Figure S5a), the HCT1i-1 line was selected for progression to the T1 generation and for creation of a double HCT-RNAi knock-down also targeting *BdHCT2* (Additional file 1: Figure S4a, see Experimental Procedures for details of construct selection and generation). Of the 14 progeny T1 lines generated, 12 exhibited moderate to strong down-regulation of HCT1 transcripts (Additional file 1: Figure S5b). The BdHCT1i-1 T1 line that was selected for further study exhibited strong down-regulation of *BdHCT1* transcripts but, as in the T0 generation, no reduction in *BdHCT2* transcript level (Fig. 3b).

Transcript levels for the target genes in stem tissues of the double knock-down T0 lines generated are shown in Fig. 3c. Although line HCT1i:HCT2i-22 exhibited the best transcript reduction, this line had poor seed yield. Line HCT1i:HCT2i-8 was therefore progressed to the T1 generation. HCT1 transcript levels were extremely low in this line (Fig. 3d), but HCT2 transcripts, although reduced, remained at around 60% of wild-type level.

### Phenotypic effects of HCT down-regulation

**Fig. 4:**
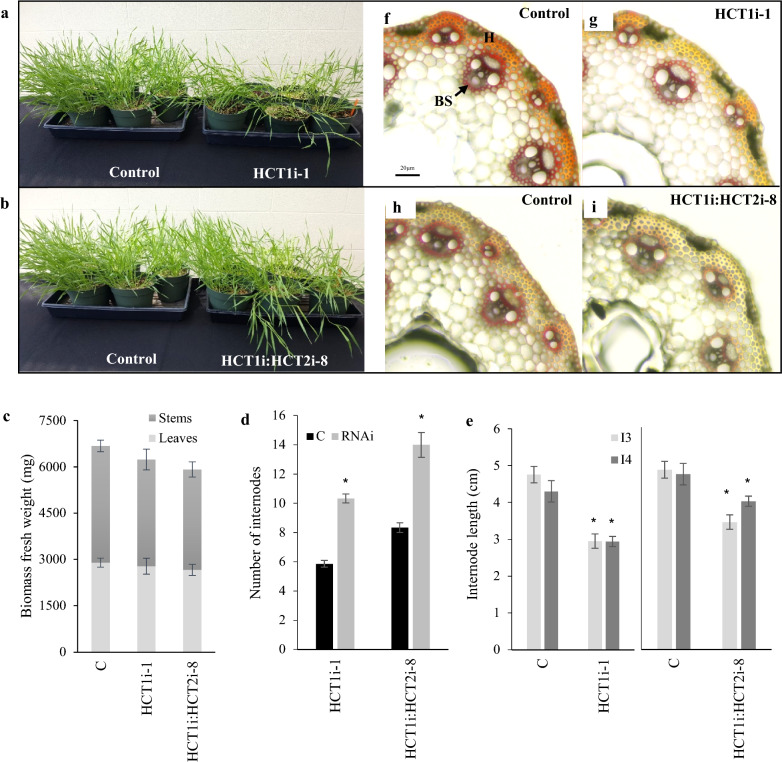
Phenotypic effects of down-regulation of HCT in *B. distachyon*. **a**, **b** Physical appearance of plants at 45 days after germination. **c**–**e** Biomass parameters of plants; **c** total above ground biomass fresh weight, **d** average number of internodes per stem, **e** Third (I3) and fourth (I4) internode lengths. **f**–**i** Phloroglucinol staining of fourth internodes. Data shown are for T1 lines, and are expressed as means of three technical replicates of 9–16 samples grouped in three biological replicates. Asterisks indicate statistically significant differences (*P* < 0.05)

Even modest down-regulation of HCT causes dwarf phenotypes in the model dicot plants *A. thaliana*, *Nicotiana benthamiana*, and *M. truncatula* [4, 9] and in the forage crop alfalfa (*Medicago sativa*) [8]. In contrast, the present HCT down-regulated *B. distachyon* plants, although showing a lodging phenotype and reduced staining by phloroglucinol of cinnamaldehydes present in the lignin in xylem, fiber, and tracheary tissues (Fig. 4a, b), exhibited only a small apparent reduction in overall stem or leaf biomass, which was significant (around 8%) only for HCT1:HCT2 in stems (Fig. 4c). Although there was a significant reduction in internode length in the HCT-RNAi plants when compared to wild-type plants at the same developmental stage (Fig. 4d), this was compensated for by a greater number of internodes (Fig. 4e).

### Lignin content and composition in HCT-RNAi lines

Lignin content and composition of T0 and T1 transgenic lines were determined by thioacidolysis (Fig. 3e–h; Additional file 1: Table S2). Surprisingly, the HCT1i-1 T0 lines showed no significant reduction in total lignin thioacidolysis yields (in some lines it was significantly increased), but a small increase in the proportion of H units (Fig. 3e). This increase in H units, which is diagnostic for HCT down-regulation [16], was more apparent in the HCT1i-1 T1 line, which also exhibited a reduction in both G and S units (Fig. 3f). The reduction in lignin content was more striking in the T0 HCT1i:HCT2i lines, but neither these nor the subsequent T1 HCT1i:HCT2i lines exhibited any greater level of H units than the up to 8% seen in the HCT1i-1 lines (Fig. 3g, h). Thus, down-regulation of both *HCT* genes in *B. distachyon* does not result in the same dramatic increase in H-lignin formation (up to 50% of total lignin in some cases) seen in dicots [4, 8, 9].

Phloroglucinol staining of stem cross sections showed that the reduced lignin levels in T1 single and double HCT knock-down lines were observed in both vascular tissues (xylem and phloem) and fibers (Fig. 4f–i).

### Saccharification efficiency in relation to cell wall composition of HCT-downregulated B. distachyon lines

To evaluate saccharification efficiencies of HCT-downregulated *B. distachyon* lines, we generated T2 progeny from T1 HCT1i-1 and HCT1i:HCT2i-8 transgenic lines, screening seedlings by PCR to ensure possession of the hygromycin resistance marker gene. The T2 plants were harvested at 45 dag along with 12 independent wild-type control plants. Lignin content and composition were also re-measured in these plants. Total lignin levels were more strongly reduced in the T2 plants (Additional file 1: Figure S6a) than in the T0 and T1 lines analyzed in Fig. 3e–h. However, as before, the increase in H-lignin was greater in the HCT1i-1 than in the HCT1i-HCT2i plants, and the proportion of H units (H/T ratio) remained well below the 50% value seen on down-regulation of HCT in some dicot plants (Additional file 1: Figure S6b,c) [8]. The S/G ratio was also significantly elevated in most of the HCT-down-regulated T2 plants (Additional file 1: Figure S6d).

**Fig. 5:**
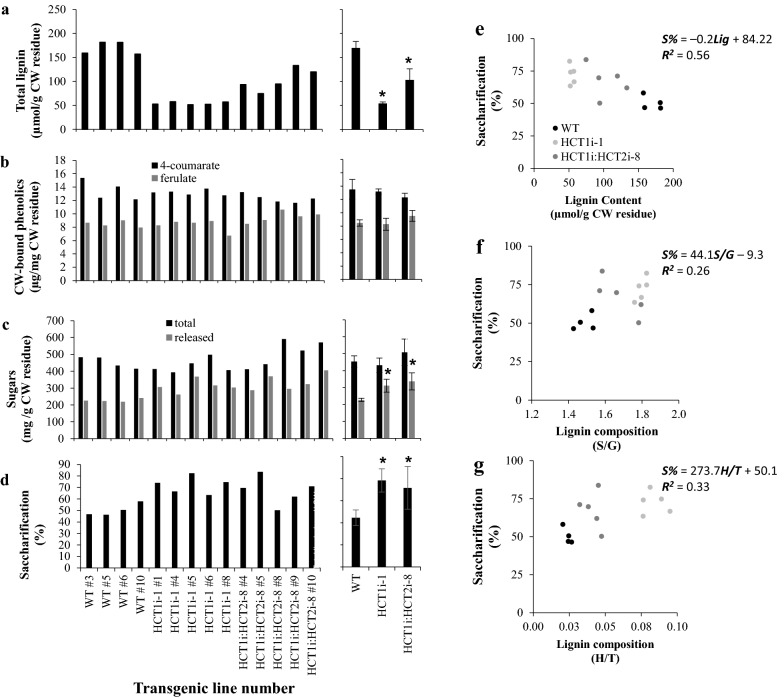
Cell wall composition and saccharification efficiency of T2 generation *B. distachyon* plants down-regulated in HCT1 or HCT1 and HCT2. **a**-**d** show data for individual lines on the left, means and standard deviations for the group on the right. **a** Total lignin as determined by thioacidolysis (see Figure S6 for monomer composition). **b** Cell wall-bound 4-coumaric and ferulic acids. **c** Total sugar content of cell wall residues in mg glucose equivalents. **d** Saccharification efficiency of cell wall residues, based on enzymatic sugar release without pretreatment relative to the total available cell wall sugar. Right hand panels show relationship between saccharification efficiency and lignin content (**e**), S/G (**f**) and H/T (**g**) monomer ratios. Asterisks indicate statistically significant differences (*P* < 0.05)

Four wild type, 5 HCT1i-1 and 5 HCT1i-HCT2i-8 T2 plants were then selected for further study. Cell wall-esterified 4-coumaric and ferulic acid levels were determined, and saccharification efficiency of extractive-free cell wall material was determined by digestion with a cocktail of cellulase and cellobiase without pretreatment [17]. Figure 5 summarizes the cell wall composition (total lignin, wall-bound phenolics and total cell wall sugar content) and saccharification efficiencies of these selected plants. Down-regulation of HCT1 or both HCT1 and HCT2 resulted in no significant change in the levels of total wall-bound coumaric and ferulic acids (Fig. 5b). Total sugar contents of cell wall residues appeared lower in some of the HCT transgenics and higher in others, but the overall change in the group of plants analyzed was not significant (*P* < 0.05) (Fig. 5c). However, there were significant increases in the amounts of sugar released (Fig. 5c) and in the corresponding calculated saccharification efficiencies of the single and double HCT-downregulated plants (Fig. 5d).

Regression analysis of various cell wall parameters against saccharification efficiency in the 15 plants tested showed an inverse correlation between lignin content and saccharification efficiency (Fig. 5e). Only weak positive correlations were seen between S/G and H/T ratios and saccharification efficiency (*R*^2^ = 0.26 and 0.33, respectively) (Fig. 5f,g).

### NMR analysis reveals only small changes in lignin composition in HCT-RNAi lines

**Fig. 6:**
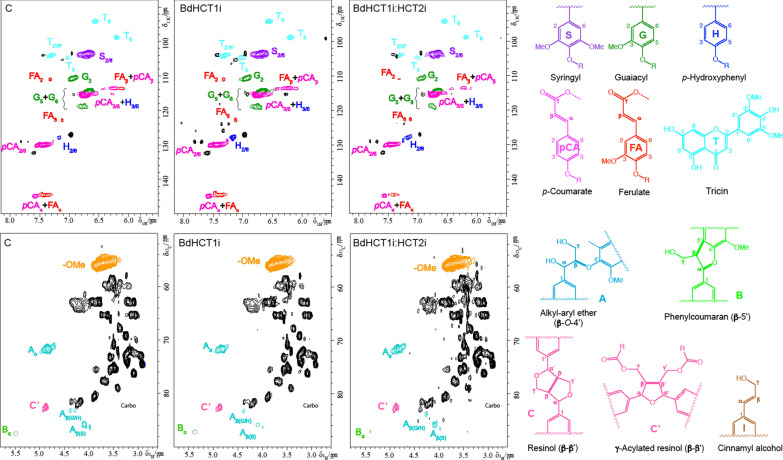
Aromatic (upper panels) and aliphatic (lower panels) subregions of short-range ^1^H–^13^C correlation (HSQC) NMR spectra of lignin-enriched cell walls from the stems of wild-type and of T2 generation *B. distachyon* lines down-regulated in HCT1 (HCT1i-1) or HCT1 and HCT2 (HCT1i-HCT2i-8). Color-coded structures are shown on the right

Next, we interrogated the monolignol composition and structure of extractive-free lignin samples isolated from the wild type and selected HCT-RNAi plants by NMR spectroscopy (see Experimental Procedures for details). We preformed two-dimensional ^1^H–^13^C heteronuclear single quantum coherence (HSQC) correlation NMR analysis on the lignin samples. Aromatic and aliphatic subregions of short-range ^1^H–^13^C HSQC NMR spectra of the extracted lignin samples are shown in Fig. 6, and summaries of the data on lignin composition (including hydroxycinnamic acid and tricin content) and linkages type are provided in Additional file 1: Table S3. The aromatic regions of the HSQC spectra of *B. distachyon* lignin showed well-resolved and strong signals from G and S lignin units along with H unit signals at lower levels. Volume integration of the well-resolved signals for lignin units can give a relative abundance of H, G, and S units in the lignin. The NMR results revealed an approximately threefold increase in H units in the single and double HCT-RNAi lines as compared to the wild type, consistent with the results of thioacidolysis (Additional file 1:Figure S6). The levels of H-units (< 11%) in HCT1i-1 and HCT2i-8 lines were still significantly lower than the combined S + G units (Additional file 1: Table S3), suggesting the limited enrichment of H units in *B. distachyon* HCT-RNAi lines. The HSQC analysis also confirmed the increase of lignin S/G ratio in HCT-RNAi lines, indicating that the increase in H units is largely at the expense of G units. Along with the signals from the monolignol (i.e., S/G/H) units, signals from the peripheral pending moieties, *p*CA and tricin, were evidently observed in the aromatic regions of HSQC spectra. In addition, although ferulates have been reported to primarily acylate arabinoxylans in the grass cell wall, HSQC spectra of the isolated *B. distachyon* lignin clearly showed the diagnostic signals of FA units in the wild type and HCT transgenic lines. The NMR analysis revealed a decreased level of lignin-bound FA units in the HCT-RNAi lines. This is in contrast with the results from the alkaline treatment analysis which demonstrated a slight (but still not significant, *P* = 0.0536) increase of FA units in the cell wall of HCT1i:HCT2i-8 double transgenic plants (Fig. 5b). It should be noted that the alkaline treatment analysis detected cell wall-bound phenolic units on both lignins and polysaccharides. The levels of coumaric acid and tricin were slightly higher in the HCT1i-1 plants, but either slightly lower or not significantly different in the HCT1i-HCT2i-8 transgenic plants as compared to the wild type (Table S3). The aliphatic regions of HSQC spectra demonstrated that β-O-4 linkages were the major linkage types between lignin monomer units along with a minor amount of phenylcoumaran (β-5) units (< 2%). The signals of resinol (β–β) units were under the noise level of NMR spectra; however, γ-acylated resinol units were observed in both the WT and HCT-RNAi lines at considerable amount (i.e., 8–10%). Compared to the wild type, the lignin in the HCT-RNAi lines had a slight decrease in the content of β-*O*-4 linkages.

### HCT down-regulation results in a small reduction in lignin molecular weight

HCT down-regulation in alfalfa leads to a large increase in H units (to approximately 70% of total monomer units) associated with a corresponding decrease (approximately 33%) in lignin molecular weight [8, 18]. The effect of HCT down-regulation on lignin molecular weight in *B. distachyon* was determined by gel permeation chromatography of acetylated lignin samples as described in Experimental Procedures. Compared to the WT (average Mol Wt 6057), the molecular weights of lignin from HCT1i-1 (4412) and HCT1i:HCT2i-8 (5460) RNAi lines were decreased by 17.5% and 9.3%, respectively (Additional file 1: Figure S7).

## Discussion

### Down-regulation of HCT in *B. distachyon*

*B. distachyon* is becoming a popular model for studies on lignin biosynthesis and engineering in grasses. Previous studies have demonstrated that down-regulation of COMT and CAD in *B. distachyon* result in altered flowering time, increased stem count and weight and decreased lignin content [19], and that around 50% of the lignin in *B. distachyon* is synthesized via deamination of L-tyrosine rather than L-phenylalanine [20]. These studies did not, however, address the paradoxical results questioning the operation of the shikimate shunt in lignin biosynthesis in grasses, namely the lack of expected effects of down-regulation of HCT or CCoAOMT in switchgrass [10, 11], and the lack of a recognizable *CSE* gene in *B. distachyon* and some other grass species [6]. In the present study, we begin to address these issues by targeting HCT for down-regulation in *B. distachyon*. Our initial hypothesis was that, based on the large increase in the proportion of H monomers in lignin and the severe growth phenotypes observed on even modest down-regulation of HCT in dicots as described above, the phenotypes observed in *B. distachyon* might be even more severe than in switchgrass in view of the lack of a CSE enzyme in *B. distachyon* that can substitute for the reverse HCT reaction [6].

In alfalfa or *A. thaliana*, blocking the shikimate shunt by down-regulation of C3´H or HCT (even partially) results in greater reductions in lignin amount than reported here, with equal or larger increases in the proportion of H units (16- to 31-fold in alfalfa and sixfold in *A. thaliana*) accompanied by highly stunted growth [8, 21, 22]. In contrast, reduced lignin levels were not observed in most T0 HCT down-regulated lines, although the T1 lines generated from event HCTi-1 showed reduction in lignin level, which was stronger in the T2 lines, reaching around threefold on average. This represents a large reduction in lignin content for this species. Reduction of lignin levels in the T1 lines selected for further analysis was accompanied by an altered plant phenotype, with lodging and more but shorter internodes in all cases, but with little reduction in total biomass. The lodging is likely the result of reduced lignin levels. Co-down-regulation of both HCT1 and HCT2 did not further increase the proportion of H units, and did not result in the dwarf phenotype observed in dicots. It is possible that the relatively low H lignin content, even in the HCT-RNAi lines with the largest lignin reduction, results from recruitment of upstream precursors to other compounds such a flavonoids; future metabolomics analyses of these lines, along with labeled precursor feeding, might help resolve this question.

On comparing the kinetics of HCT1 and HCT2 from *B. distachyon* with those of the corresponding enzymes from switchgrass, *A. thaliana* and *M. truncatula* (which all possess the CSE reaction), BdHCT2 exhibited among the more favorable kinetics for the conversion of caffeoyl shikimate to caffeoyl CoA. Further comparisons indicate that the reverse reaction for *B. distachyon* HCT2 is much more efficient than for HCT1 and HCT6 from *Populus trichocarpa*, which show Kcat/Km values 300 to 400 times lower for the reverse reaction [23], consistent with the operation of CSE in poplar [6, 24]. The Km values for the forward reactions reported for the *B. distachyon* HCTs in the present work are similar to those for the enzymes from poplar [23], but much lower than those reported for HCTs from tobacco and *Cynara cardunculus*, which prefer caffeoyl CoA as substrate for transesterification with shikimate [25, 26]. In *M. truncatula* and switchgrass, the ratio of the forward to the reverse HCT reaction is similar for both forms of the enzyme, whereas in *B. distachyon*, the ratio for HCT2 is around 5 times lower than for HCT1. The reverse HCT reaction could therefore function in *B. distachyon* to allow the shikimate shunt to operate in the absence of CSE. That said, we have not been able to demonstrate a significant reverse HCT reaction in crude stem protein extracts from *B. distachyon*.

The absence of CSE itself could further favor the reverse HCT reaction in *B. distachyon*. Although we did not measure the kinetics for conversion of caffeoyl CoA to caffeyl shikimate for the *B. distachyon* HCTs, this reaction is preferred to the reverse reaction for both switchgrass [27] and poplar [23] HCTs. If the reaction converting caffeoyl CoA to caffeoyl shikimate were more efficient than the reverse reaction such that the reverse reaction does not occur, and CSE were also present, in the absence of some form of channeling to facilitate *O*-methylation of caffeoyl CoA there is the potential for a futile cycle that would hydrolyze ATP. Why this does not happen is not clear, but the absence of CSE would allow the accumulation of higher levels of caffeoyl shikimate to drive the reverse reaction; efficient capture of the generated caffeoyl CoA by CCoAOMT may also assist. Clearly there is more to learn about the biochemical consequences of possession or lack of possession of CSE.

A hydroxycinnamoyl CoA: quinate hydroxycinnamoyl transferase (HQT) is involved in the biosynthesis of chlorogenic acid (caffeoyl quinic acid, CGA), but the intermediacy of CGA in lignin biosynthesis remains controversial [28]. Few examples of the reverse HQT reaction to convert CGA to caffeoyl CoA have been reported, with no data on kinetic parameters. Switchgrass does not possess a “classical” HQT enzyme, and the two *HCT* genes encode enzymes with strong preference for shikimate, not quinate [27]. An “HCT-like” enzyme from switchgrass was, however, shown to possess HQT activity, and orthologs of the gene encoding this protein are found in the *B. distachyon* genome. However, as this HCT-like enzyme did not operate in the reverse direction to generate caffeoyl CoA [27], it seems unlikely that there is a pathway from CGA to lignin in *B. distachyon*.

### Factors responsible for cell wall recalcitrance in *B. distachyon*

HCT down-regulation provides the greatest increase in saccharification efficiency in a comparison of various lignin-modified alfalfa lines [29]. HCT has been avoided as a target for lignin modification for agronomic improvement because of the associated negative growth effects. In other published reports of lignin modification in grasses, down-regulation of cinnamyl alcohol dehydrogenase resulted in an improvement of saccharification efficiency (after mild alkali pretreatment) of approximately 45% in *B. distachyon*, [30], and down-regulation of CCoAOMT, F5H or COMT in sugarcane resulted in variable results, with maximum improvements of no more than 45% [31]. These values are similar to the improvement in saccharification efficiency without pretreatment observed for HCT down-regulated *B. distachyon* in the present work.

In the case of HCT-antisense alfalfa, an approximately 50% reduction in total extractable HCT activity results in lignin composition (H:G:S) ratio changes from approximately 4:63:33 in wild-type plants to approximately 69:16:15 in HCT antisense lines [8], and an increase in saccharification efficiency without pretreatment of approximately 2–threefold [29]. The high proportion of H residues in the lignin leads to a decrease in its mean degree of polymerization (by approximately 33%, from 6000 to 4000), resulting in easier solubilization of lignin during acid pretreatment [18]. The most extreme lignin monomer ratio observed in the present *B. distachyon* HCT down-regulated lines (of approximately 10:32:58, from a wild-type value of 2:40:58) is small in comparison, and is associated with a smaller reduction in average lignin molecular weight of around 13% (from 6057 to 5460). Nevertheless, it is associated with a 50% increase in saccharification efficiency without pretreatment. Furthermore, in HCT antisense alfalfa there is a nearly sixfold decrease in total lignin, whereas the decrease in the *B. distachyon* HCTi lines is at most from 2- to 3-fold. In both *B. distachyon* and alfalfa, the increase in H units is primarily at the expense of G units, with the S/G ratio increasing in both cases. The present NMR analyses show some additional features that, together with the changes in lignin content, S/G ratio and molecular weight, might contribute to the improved saccharification of the HCT-RNAi *B. distachyon lines*. There is also a decrease in lignin-associated ferulate level in HCT-RNAi *B. distachyon* lines. Grass lignins contain ferulate 4-*O*-β- and 8-β-coniferyl alcohol cross-coupled structures that represent linkage sites to polysaccharides [32, 33], and a reduced level of these could contribute to the improved saccharification efficiency in the present work. It should be noted that there were no changes in non-lignin attached cell wall-esterified ferulate in the HCT-RNAi *B. distachyon* lines; down-regulation of an acyltransferase from the same family as HCT reduced levels of feruloylated arabinoxylan in S*etaria viridis*, with a 45–50% increase in saccharification efficiency [34]. The lack of effect of HCT down-regulation on the levels of γ-acylated resinol units is consistent with the effects of down-regulation of C3^′^H in rice [35].

### Parallel pathways to G-lignin in *B. distachyon*?

The shikimate shunt is the currently accepted pathway for introduction of the 3-hydroxyl group during monolignol biosynthesis. The phenolic esters of shikimate in grasses could also be signaling molecules, or act as antioxidants, UV screens, or precursors to other molecules such as chlorogenic acids or even biopolymers like suberin. We here show that HCT2 from *B. distachyon* is more efficient than HCT from *A. thaliana* in catalyzing the conversion of caffeoyl shikimate to caffeoyl CoA, perhaps explaining how the shikimate shunt functions in the absence of a CSE enzyme in *B. distachyon*. This does not, however, explain the differences in growth phenotype on down-regulation of HCT in the two species. We have recently demonstrated the operation of an alternative pathway to monolignols involving direct hydroxylation of coumaric acid to caffeic acid by a soluble peroxidase (C3H) [7]. Complete loss of function of C3H appears to be lethal in both *B. distachyon* and *A. thaliana* [7]. We have suggested that the C3H-requiring “acids pathway” to caffeic and ferulic acids is more efficient in monocots than in dicots, providing an alternative route to 3-*O*-methylated lignin and lignan precursors which might be important for growth and development, in addition to cell wall polymer biosynthesis. Operation of this pathway might explain why down-regulation of HCT has less impact on growth in *B. distachyon* than in dicot model species. Genetic analysis is in progress to test this hypothesis. However, operation of the “acids pathway” does not substitute totally for the shikimate shunt; the relative activities of the shikimate shunt and acids pathways might change depending on environmental conditions, perhaps associated with the higher activity of HCT2 than HCT1 for the reverse reaction to generate caffeoyl CoA. The absolute expression level of HCT2 in wild-type plants was quite variable. HCT2 was expressed from 5- to 20-fold lower that HCT1 in stems of the wild-type plants analyzed initially, whereas its expression was equal to or more than that of HCT1 in the wild-type plants grown with the T1 and double HCT-RNAi lines. All the plants in these studies were grown in the same growth chamber with identical temperature and light settings, but humidity was not controlled and may have varied considerably. We did not see this relative variability in transcript levels for other monolignol pathway genes within the same biological materials, suggesting that HCT2 expression might be highly sensitive to environmental factors. Further studies will be necessary to determine whether the relative operation of the shikimate shunt is affected by environmental conditions.

### Biotechnological implications of HCT down-regulation in grasses

Reducing lignin content has found broad application in the development of improved bioenergy feedstocks and forages [29, 36–39]. Whereas down-regulation of HCT, although leading to large improvements in cell wall sugar release in some species, is problematic in dicots because of its negative yield impacts, this is not so in *B. distachyon* (present data) or switchgrass [10]. The impacts on cell wall recalcitrance in *B. distachyon* are most likely manifested through changes in HCT1 expression, because double knock-down lines exhibited similar saccharification efficiencies to lines down-regulated in HCT1 alone. It was recently shown that HCT down-regulation can be effective for improving digestibility in field-grown alfalfa [40], although lines had to be carefully selected to ensure good agronomic performance, and this meant that the potentially higher digestibility of the more strongly down-regulated lines could not be exploited. In *B. distachyon*, HCT down-regulation results in significant increases in saccharification efficiency without loss in biomass.

## Conclusions

Our results identify HCT as a promising target for cell wall engineering in *B. distachyon* and potentially other grass species. We suggest that this arises from amelioration of negative effects of HCT down-regulation on plant growth through redundancy to by-pass the shikimate shunt in grasses, and a greater sensitivity of cell wall recalcitrance to increased proportion of H monolignol units, lignin-associated ferulate, increased S/G ratios and decreased lignin molecular weights.

## Methods

### Chemicals

4-Coumaroyl CoA and caffeoyl CoA were synthesized as described previously [41]. Shikimic acid was purchased from Sigma-Aldrich (St. Louis, MO). 4-Coumaroyl shikimate and caffeoyl shikimate were kindly provided by Dr. John Ralph (University of Wisconsin-Madison).

### Plant material, transformation and growth conditions

Seeds of *B. distachyon* diploid inbred line Bd21-3 were used to establish plants. Calli and regenerated plantlets were grown under a 16-h light: 8-h dark photoperiod at constant 25 °C temperature. Wild-type plants grown from seeds and RNAi plants were grown in the greenhouse with 25/22 °C day/night and 14 h daylight supplemented with PAR (photosynthetically active radiation) lights when the range was out of 40–120 mol photons/m^2^/sec during the day period. To create single gene knock-down lines, *Agrobacterium tumefaciens* strain EHA105 containing the pANIC8A vector [15] (Additional file 1: Figure S4b) with RNAi inverted repeats was used as transformation agent in wild-type calli. In the case of double HCTi lines, calli generated from caryopses of single transgenic line HCT1i-1 were re-transformed with the HCT2i construct. Since the selection marker was the same, we used qPCR to determine the down-regulation of both HCT1 and HCT2 transcripts in the lines generated; lines that had no down-regulation of HCT2 were discarded. Caryopses of *B. distachyon* were sterilized in 10% bleach for 10 min, washed four times in sterile water for 1 min and transferred to CIM (callus induction medium) for 4 weeks. Developing calli were transferred to fresh CIM every 2 weeks for 6 weeks in the dark. Calli were then transferred to the light in regeneration medium until plantlet development, and finally transferred to MS (Murashige and Skoog) growing medium as previously described [20]. Plants with transgenes were selected using hygromycin B (50 mg/l) in CIM, regeneration medium and MS medium after transformation. Plants were grown in the greenhouse in half-gallon pots containing Sungro® Metro Mix 360. T0 (primary transformant, regenerant zero) plants were individual lines generated from calli of initial transformations; T1 and T2 plants were grown from seeds of individual T0 and T1 lines, respectively.

### Phylogenetic analysis

Protein sequences of HCT in *B. distachyon* were Blast searched using the National Center for Biotechnology Information (NCBI) GeneBank database. Selected amino acid sequences with high homology were aligned using Geneious 10.0.9 software (Biomatters Ltd.) under clustalW alignment and blosum cost matrix. The phylogenetic tree was built by PHYML plugin [42] using JTT substitution model and 1000 bootstraps replicates optimized by topology, length and rate with the BEST (NNI and SPR) search. Description of species used and accession numbers are given in Fig. 2.

### Histochemistry

Transverse internode sections of wild-type and RNAi lines (45 days after germination) were sliced (100 µm thick) using a HM 650 V Vibrating Blade Microtome (Thermo Fisher) and stained with phloroglucinol–HCL (3% (w/v) phloroglucinol in ethanol:12 N HCL in a 1:2 ratio). Images were taken with an EVOS™ XL Core Imaging System (Thermo Fisher).

### RNAi-mediated suppression of target genes

Conserved coding regions of the BdHCT family were analyzed to determine the target RNAi fragments. Analysis of gene sequences and primers was made using Geneious 10.0.9 software and SnapGene software (GSL Biotech LLC) for vector assembly. The RNAi fragments cloned in the pANIC8a vector using the Gateway® cloning technology (Life Technologies) were: HCT1 (259 bp), HCT2 (360 bp).

### RNA extraction and real-time qPCR

For initial time course experiments, roots, leaves, stems and fruits of 15 and 45 dag plants were chosen for total RNA extraction with Trizol® (Thermo Fisher). In the case of T0 and T1 single and double mutants, internodes 3–5 from 45 dag plants were used. Total RNA (3 µg) quantified by a NanoDrop ND-1000 spectrophotometer (NanoDrop Technologies) was treated with Invitrogen™ TURBO DNA-free kit (Fisher scientific) and cDNA was extracted with the High-Capacity cDNA Reverse Transcription Kit (Thermo Fisher). 20-Fold dilutions of cDNA were used as templates using a QuantStudio 6 Flex Real-Time PCR System and Power SYBR Green Master Mix (Thermo Fisher). In the case of T0 populations, three biological replicates and three technical replicates were used for analysis. For T1 populations, every biological replicate was composed of 4 samples, and 3 technical and 3 biological replicates were used for analyses as described previously [43]. Primers for amplification are shown in Additional file 1: Table S4. The regions chosen for transcript analyses were out of the RNAi target region. *B. distachyon* tubulin (XM_003560329.1) was used as a housekeeping gene for calculating relative expression.

### Cloning of HCT cDNAs

Full length HCT cDNA sequences were found in the public databases Phytozome (https://phytozome.jgi.doe.gov/pz/portal.html) and The Arabidopsis Information Resource (TAIR; https://www.arabidopsis.org/) after a search using the protein sequences of AtHCT, PvHCT1 and PvHCT2 as queries for BLAST (BLASTP) analysis. Electronic sequences were used for primer design (Additional file 1: Table S4) to clone the coding region of the targeted genes. Leaf or stem tissues of *B. distachyon*, *A. thaliana* and *M. truncatula* were collected to isolate total RNA using Trizol reagent (Invitrogen, Carlsbad, CA) following the manufacturer’s instructions. AtHCT, BdHCT1, BdHCT2, MtHCT1 and MtHCT2 coding regions were amplified by RT-PCR using forward and reverse primer pairs (Additional file 1: Table S4) using the SuperScript III First-Strand System for RT-PCR Kit (Thermo Fisher Scientific, https://www.thermofisher.com). The cDNAs were cloned into pENTR-D Topo and then into pDEST17 Vector (Thermofisher Scientific) by LR recombination reaction.

### Expression of HCTs in *E. coli*

pDEST17-HCT constructs were introduced into *E. coli* Rosetta strain cells. These were cultured at 37 °C and the heterologous protein expression started by addition of isopropyl 1-thio β-galactopyranoside (IPTG) to a final concentration of 0.5 mM when the culture OD_600_ reached between 0.6 and 0.9. The cultures were incubated at 16 °C for 18–20 h and the cells collected and frozen at − 80 °C. Purification of heterologously expressed proteins was performed as previously described [27]. The percentage purity of recombinant HCT proteins was determined from the SDS-PAGE images (Additional file 1: Figure S1) using the software Image J (https://imagej.nih.gov/ij/download.html). These percentage purities and total protein contents of the recombinant preparations determined by Bradford assay were used to estimate the final amounts of HCT proteins.

### Assay of HCT enzyme activities and determination of kinetic parameters

HCT enzyme assays towards 4-coumaroyl CoA (“forward reaction”) were perform with 10 or 20 ng of recombinant proteins or 4 to 7 µg of crude plant protein extracts in a reaction solution of 100 mM sodium phosphate buffer pH 7.5, 500 µM shikimic acid and 500 µM dithiothreitol (Roche, Madison, WI) in a final volume of 100 µL. The 4-coumaroyl CoA concentration was 50 µM for assays with crude extracts, and varied from 5 to 100 µM to determine kinetics of recombinant enzymes. HCT enzyme assays towards caffeoyl shikimate (“reverse reaction”) were carried out with 50 ng of recombinant proteins or 13 to 17 µg of plant crude protein extracts in a reaction solution of 100 mM sodium phosphate buffer pH 7.5, 500 µM Coenzyme A and 500 µM dithiothreitol (Roche, Madison, WI) in a total volume of 100 µL. The caffeoyl shikimate concentration was 50 µM for assays with crude extracts, and varied from 20 to 400 µM to determine kinetics of recombinant enzymes. Reactions were terminated by addition of 10 µL of glacial acetic acid and products were analyzed by HPLC as previously described (Escamilla-Trevino et al*.* [27]) Products were quantified by measuring peak areas and converting to units of quantity using calibration curves that were constructed with authentic standards of each product.

### Determination of lignin content and composition

Cell wall residues were prepared from about 200 mg of ground frozen stem internodes by sequential extraction with methanol (100%), chloroform:methanol (2:1) and methanol (100%) again. Thioacidolysis was performed using 6 or 10 mg of cell wall residue samples incubated with 3 ml of 0.2 M BF_3_ etherate in an 8.75:1 dioxane/ethanethiol mixture [44, 45]. Lignin-derived monomers were identified by gas chromatography mass spectrometry (GC/MS), and quantified by GC as their trimethylsilyl derivatives. GC/MS was performed on a Hewlett–Packard 7890A gas chromatograph with a 5975C series mass selective detector (column: Agilent DB-5 ms, 60 m × 0.25 mm × 0.25 μm film thickness). Mass spectra were recorded in electron impact mode (70 eV) with 60–650 m/z scanning range.

### Lignin isolation and purification

Extractives were removed from dried *B. distachyon* stem samples by extraction with toluene:ethanol (2:1, v/v) for 8 h, followed by extraction with acetone for 4 h; the samples were then air dried. The extractives-free samples (1.2–1.6 g) were then ground in a Planetary Ball Mill PM 100 for 2 h with a milling cycle consisting of a 5-min milling period at 600 rpm, followed by a 5-min pause to avoid overheating. The ball-milled grass material was transferred to 50 mL plastic sample tubes together with cellulase from *Trichoderma* sp. (10 KU; Sigma-Aldrich, St. Louis, MO) dissolved in 50 mM acetate buffer (pH 5.0), with substrate loading of 30 mg/mL. The enzyme load was 45 mg/g biomass. Enzymatic hydrolysis was conducted at 37 ℃ for 48 h with mixing at 160 rpm. After cellulase treatment, the mixture was centrifuged and the liquid removed. The same cellulase treatment was repeated twice. The enzyme-hydrolyzed lignin residues were purified by washing with fresh water to remove monosaccharides, oligosaccharides and enzymes for three times, then freeze-dried. The residue was then extracted with dioxane-water (96% v/v) for 24 h and the process was repeated once with fresh dioxane-water mixture. The extracted mixture was centrifuged, and the supernatants were collected and combined. The obtained liquid was evaporated to remove dioxane and water using a rotary evaporator (< 45 °C) and the residues were freeze-dried to obtain the lignin samples for further analysis.

### Analysis of saccharification efficiency

Ten mg of extractive-free stem cell wall material was mixed with 0.5 mL of 72% sulfuric acid. Samples were incubated at 30 °C for 1 h on a shaker at 100 rpm and were then diluted with 14 mL of water and heated in an autoclave at 120 °C for one hour. Hydrolysates were collected by centrifugation, and stored at − 20 °C prior to total sugar analyses as described below.

Enzymatic saccharification was performed according to the analytical procedure of the National Renewable Energy Laboratory (https://www.nrel.gov/docs/gen/fy13/42618.pdf). Ten mg of cell wall residues were hydrolyzed with a mixture of Celluclast (cellulase from *Trichoderma reesei*) and Cellic CTec2 (a blend of cellulases, β-glucosidases and hemicellulase) (SAE0020, Sigma, St Louis, MO, USA) in 10 mL sodium citrate buffer (0.1 M, pH 4.8). The enzyme cocktail was obtained by mixing equal volumes of 2 mL Celluclast and Cellic CTec2. The enzyme loadings were 10 FPU per g cell wall residue. Enzyme blanks and Whatman #1 filter paper (10 mg) were digested alongside the samples. Hydrolysis of filter paper was always more than 95%. Samples were incubated in the dark at 50 °C for 72 h with shaking at 100 rpm. The antibiotics tetracycline (10 mg/mL) and cycloheximide (10 mg/mL) were added to the mixture to avoid bacterial contamination.

Total sugars were analyzed spectrophotometrically using the phenol–sulfuric acid method [46]. Determination of mg glucose equivalents per g CWR was obtained based on the glucose standard curve. Original mixtures were diluted (1/5 for total sugars and 1/8 for released sugars) for colorimetric determination. Absorbance values from released sugars were corrected for the enzyme control background. Saccharification efficiency (%) was determined as the proportion of sugars released by enzymatic hydrolysis from the total amount of sugars present in the samples.

### Determination of cell wall-bound phenolics

Twenty milligrams of cell wall residues were used for analysis of the esterified cell wall-bound phenolics ferulic and coumaric acids using alkaline hydrolysis (2 M NaOH, 37 °C, 5 h). Briefly, after acidification with 6 M HCl, the aqueous phase (pH = 2.0) was extracted three times with 1.6 ml of ethyl acetate. The ethyl acetate extracts were pooled and dried under nitrogen, resuspended in methanol, and analyzed on a reverse-phase C18 column (Spherisorb 5μ ODS2, Waters) by HPLC. The separation of ferulic acid and coumaric acid was performed with a step gradient using 1% phosphoric acid as stationary phase (A) and acetonitrile as mobile phase (B): 0–5 min 100% A; 5–10 min 95% A, 5% B; 10–25 min 90% A, 10% B; 25–30 min 83% A, 17% B; 33–35 min 77% A, 23% B; 55–59 min 50% A, 50% B. Quantification was done using calibration curves with authentic standard compounds.

### NMR analysis

NMR spectra were acquired on a Bruker Avance III HD 500-MHz spectrometer equipped with a double resonance Prodigy cryoprobe with gradience in Z-direction (Bruker BBO-H&F BBO-HD-05 Z). The lignin sample was dissolved in DMSO-d6 and a standard Bruker heteronuclear single quantum coherence (HSQC) pulse sequence was used with the following acquisition parameters: spectra width 12 ppm in F2 (^1^H) dimension with 2048 time of domain, 220 ppm in F1 (^13^C) dimension with 256 time of domain, a 1.5-s delay, a ^1^J_C–H_ of 145 Hz, and 64 scans. The central DMSO solvent peak (^13^C/^1^H at 39.5/2.49) was used for chemical shift calibration. Assignments of lignin compositional subunits and interunit linkage were based on reported contours in HSQC spectra. The relative abundance of signals in lignin HSQC NMR spectra was estimated using volume integration of contours in spectra.

### Determination of lignin molecular weight

The molecular weight of lignin samples was measured by gel permeation chromatography (GPC) after acetylation. Dry lignin was dissolved in a mixture of acetic anhydride/pyridine (1:1 v/v) and stirred at room temperature for 24 h. Ethanol was then added to the reaction mixture and then removed with a rotary evaporator. The addition and removal of ethanol was repeated until all traces of acetic acid were removed from the sample. The samples were dissolved in tetrahydrofuran and analyzed on a PSS-Polymer Standards Service (Warwick, RI, USA) GPC/SEC 1200 system equipped with three Waters Styragel columns (HR0.5, HR3 and HR5, 4.6 × 300 mm) and a UV detector (270 nm). Tetrahydrofuran was used as the mobile phase and flow rate was 0.5 mL/min. Standard narrow polystyrene samples were used for establishing the calibration curve.

### Statistical analysis

Values were analyzed under SPSS Statistics 24 (IBM) comparing means of paired samples with *t*-test and one-way ANOVA with LSD comparisons (*P* < 0.05). For all experiments, means (± SE) of T0 lines were calculated from three technical replicates and means (± SE) of T1 lines were obtained from three technical replicates of 9–12 samples grouped in three biological replicates. Means of wild-type controls were averages of 9–16 plants unless otherwise stated. Asterisks (*) indicate statistically significant results at *P* < 0.05, or as indicated in legends.

## Supplementary Information

**Additional file 1: Figure S1.** HCT reactions in crude protein extracts and expression of recombinant HCTs in *E. coli.* **Figure S2.** Phylogenetic and structural analysis of the BAHD family of plant acyltransferases. **Figure S3.** Lignin deposition and organ-specific expression of HCT in wild-type *B. distachyon*. **Figure S4.** Construction of RNAi vectors for down-regulation of Brachypodium *HCT* genes. **Figure S5.** HCT1 and HCT2 transcripts in T0 transgenic plants in which HCT1 had been targeted by RNA interference. **Figure S6.** Lignin content and composition in T2 generation *B. distachyon* lines downregulated in HCT1 or HCT1 and HCT2. **Figure S7.** Determination of lignin molecular weight by gel-permeation chromatography*.* **Table S1.** Lignin content and composition of internodes 5 and 8 of *B. distachyon* stems harvested at 45 days after germination. **Table S2.** Individual S:G and H:total lignin monomer ratios of both single and double *B. distachyon* HCT-RNAi lines from T0 and T1 generations. **Table S3.** Lignin composition and linkage types as determined by NMR analysis. **Table S4.** Primers used in the present work.

## Data Availability

All data generated or analyzed during this study are included in this published article and its supplementary information files.
