# Supplementary material for: Targeting hydroxycinnamoyl CoA: shikimate hydroxycinnamoyl transferase for lignin modification in Brachypodium distachyon
Source: Biotechnol Biofuels. 2021 Feb 27;14:50. doi: 10.1186/s13068-021-01905-1 (PMC7913460; doi:10.1186/s13068-021-01905-1)
Supplement: Supplementary file 1 — Additional file 1: Figure S1. HCT reactions in crude protein extracts and expression of recombinant HCTs in E. coli. Figure S2. Phylogenetic and structural analysis of the BAHD family of plant acyltransferases. Figure S3. Lignin deposition and organ-specific expression of HCT in wild-type B. distachyon. Figure S4. Construction of RNAi vectors for down-regulation of Brachypodium HCT genes. Figure S5. HCT1 and HCT2 transcripts in T0 transgenic plants in which HCT1 had been targeted by RNA interference. Figure S6. Lignin content and composition in T2 generation B. distachyon lines downregulated in HCT1 or HCT1 and HCT2. Figure S7. Determination of lignin molecular weight by gel-permeation chromatography. Table S1. Lignin content and composition of internodes 5 and 8 of B. distachyon stems harvested at 45 days after germination. Table S2. Individual S:G and H:total lignin monomer ratios of both single and double B. distachyon HCT-RNAi lines from T0 and T1 generations. Table S3. Lignin composition and linkage types as determined by NMR analysis. Table S4. Primers used in the present work. [file 13068_2021_1905_MOESM1_ESM.pdf]

Figure S1. HCT reactions in crude protein extracts and expression of recombinant HCTs in *E. coli.*
(**a**) HCT forward and reverse reaction in crude extracts from monocot and dicot plants. (**b**) SDS-PAGE analysis of semi-pure recombinant HCTs and table of its purity. Gel: The bands corresponding to recombinant HCTs are inside the rectangle on the gel. M, Molecular mass markers; At, AtHCT; Bd1, BdHCT1; Bd2, BdHCT2; Mt1, MtHCT1; Mt2, MtHCT2; Pv1, PvHCT1; Pv2, PvHCT2.

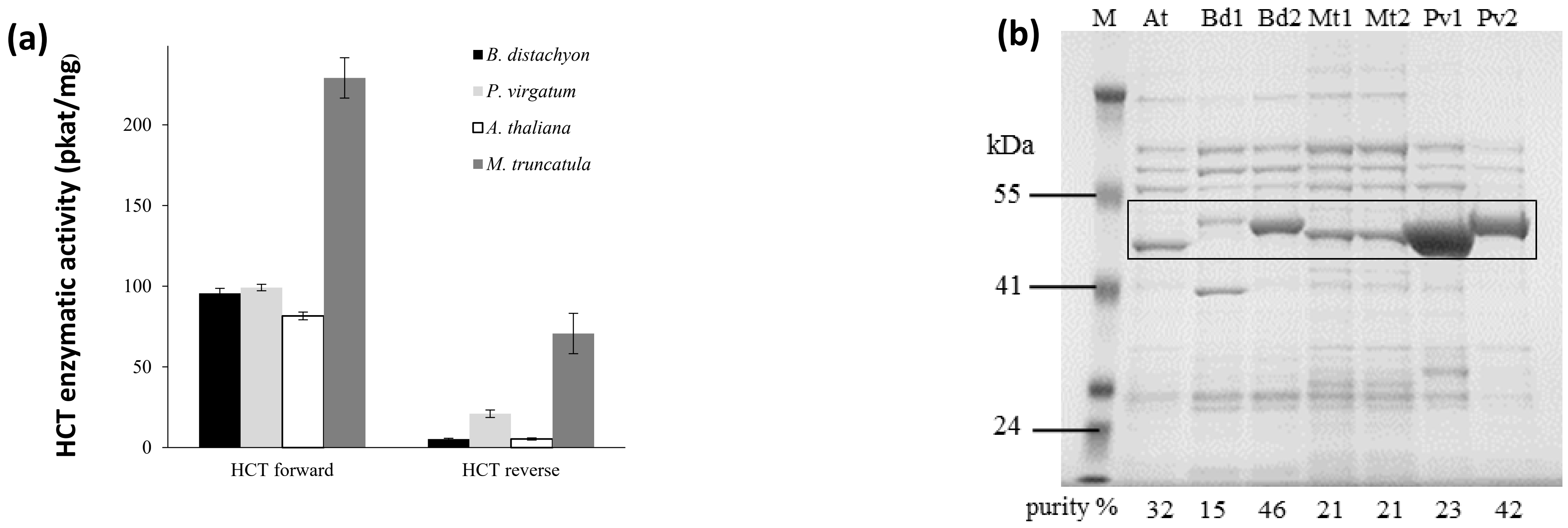

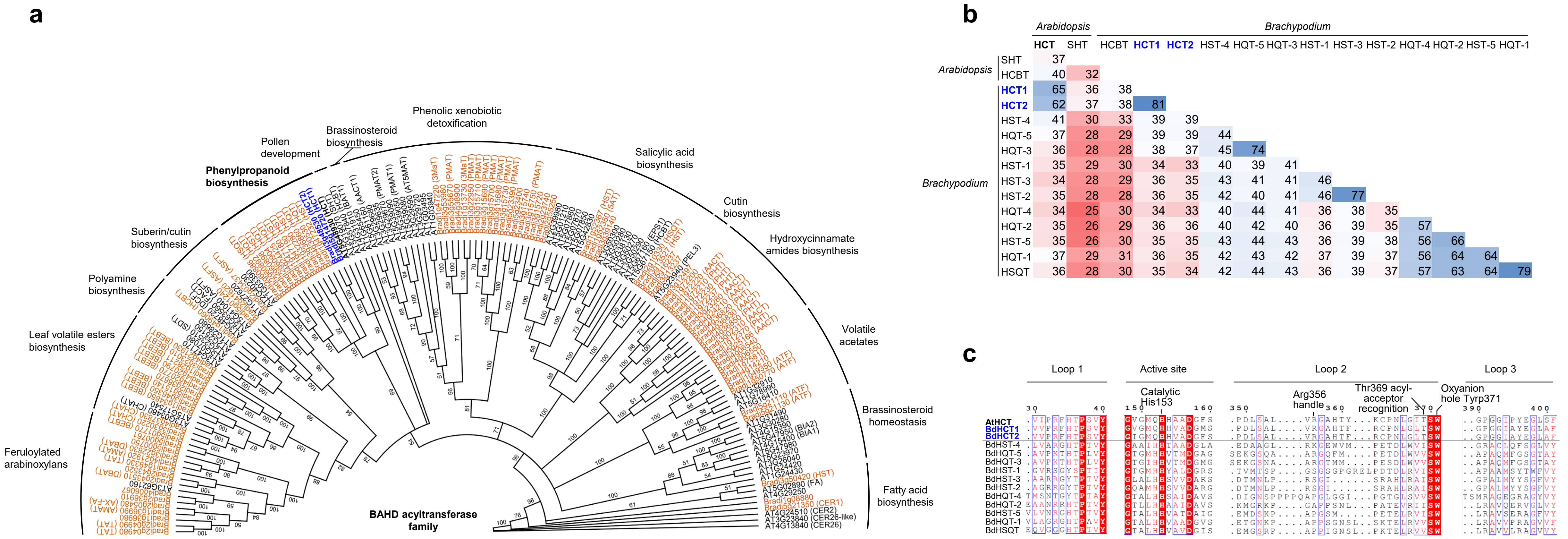

Figure S2. Phylogenetic and sequence analysis of the BAHD family of plant acyltransferases. (**a**) Phylogenetic analysis and multiple functions of the BAHD family of plant acyltransferases. (**b**) Identity of HCT-like proteins in *Arabidopsis* vs *Brachypodium* (**c**) Catalytic residues [12] in active sites of HCT and HCT-like proteins in *Brachypodium.* The substrate preference of *Brachypodium* HCTs for shikimate (HST), quinate (HQT) or both (HSQT) shown here are based on data from Phytozome v12.1.

Figure S3. Lignin deposition and organ-specific expression of HCT in wild-type *B. distachyon*. (**a**) Stem internode sections in wild type plants. Sections were taken progressively from internode one (I-1, oldest) to internode eight(I-8, youngest). Internodes 3, 4 and 5 were considered the most informative for lignin analysis in the current study. (**b**) Tissue-specific expression of HCT1 and HCT2 as displayed in the Brachypodium gene expression atlas (link). (**c**) Transcript levels of HCT1 and HCT2. Tissues analyzed were roots (R), stems (St), and leaf (Lf) at 15 and 45 days after germination (dag), and seeds (Sd) at 45 dag. Transcript levels were measured by qPCR, and are expressed relative to the housekeeping gene Bd-tubulin.

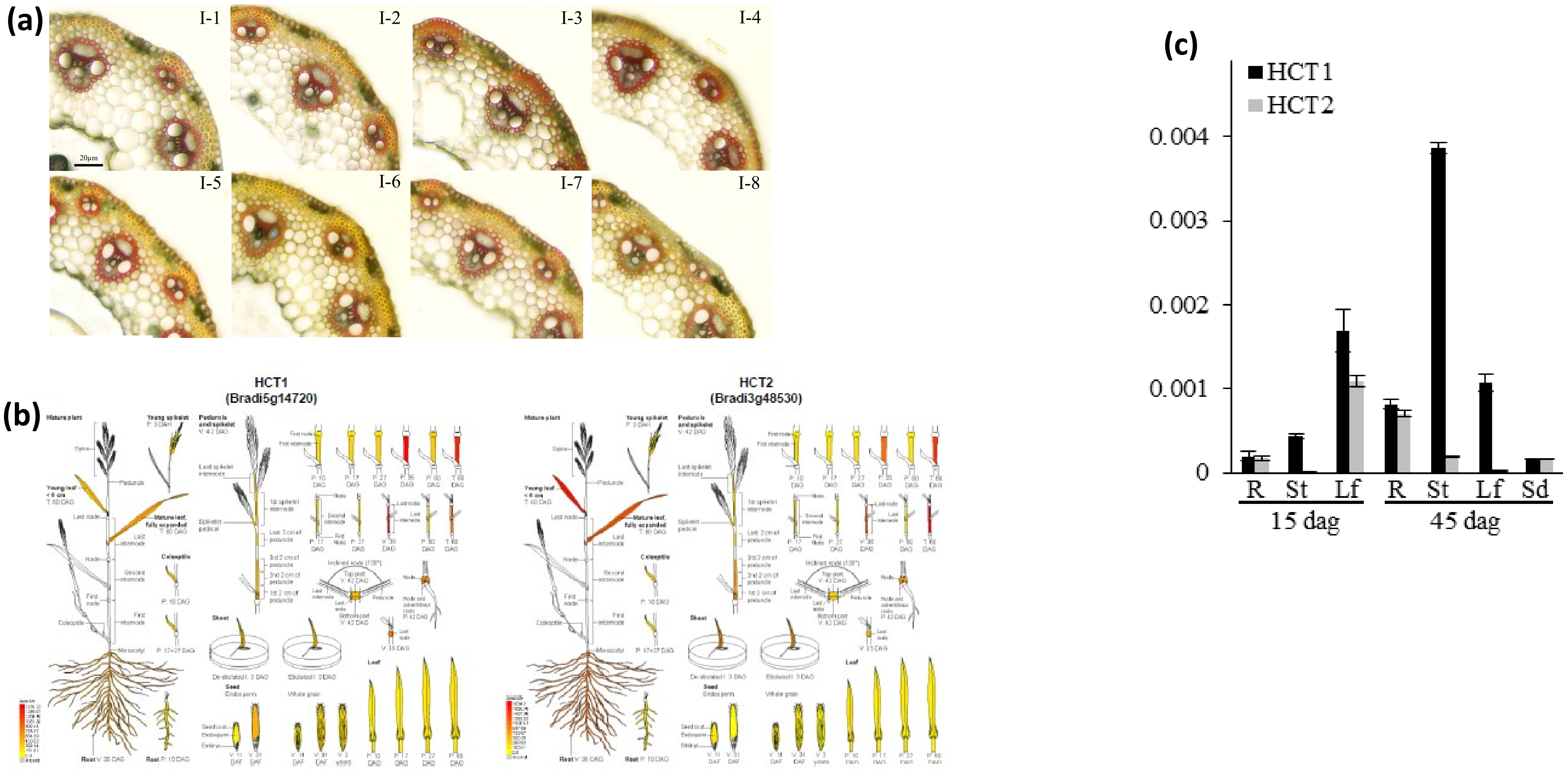

Figure S4. Construction of RNAi vectors for down-regulation of Brachypodium *HCT* genes. **(a)** Map fragment of the custom pANIC 8A-HCTi vector and the interference sequences (HCT1i and HCT2i) used to target the mRNAs. **(b)** Original pANIC 8A vector. For abbreviations see [15].

(a)

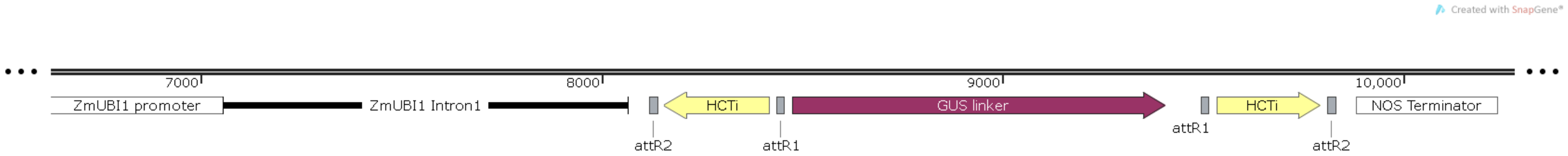

HCT1i>TGGATTACCTGGAGCTGCAACCGGATTTATCAGCATTGGTTCGAGGAGCTCACACATTCAGGTGCCCTAACCTCGGTCTCACCAGCTGGGTGCGCCTGCCGATCCACGATGCTGATTTTGGTTGGGGACGA
CCGGTGTTCATGGGTCCTGGAGGCATTGCATACGAGGGGCTGGCGTTCGTCATCCCGAGCTCGAGTAAGGATGGCAGCTTATCCATCGCTATCTCGTTGCAGGCCGAGCACATGGAGAAGTTCCGGAAG

HCT2i>GACTCGGAGCCGCCGCAGGCCATCCTCGCGGGCGCCAAGCCGGACCAACCCCCCGCGGCCGTGGACATCTTCAAGCTCTCCCGCTCCGACCTGGGCCGCCTCCGCGCCCAGCTCCCCACGGGGGAAGGCG
CGCCCCGGTTCAGCACCTACGCGGTGCTCGGCGCGCACGTGTGGCGTTGCGCGTCGCTGGCCCGCGGGCTGGCGCCCGAGCAGCCGACGAAGCTGTACTGCGCCACGGACGGGCGCCAGCGGCTGCTGCCGCCGC
TCCCGGAGGGTTACTTCGGCAACGTCATCTTCACGGCCACGCCGCTCGCCGAGGCCGGCAAGGTGATCGCCTCGCTGGCGGACGGCGCGAACACG

(b)

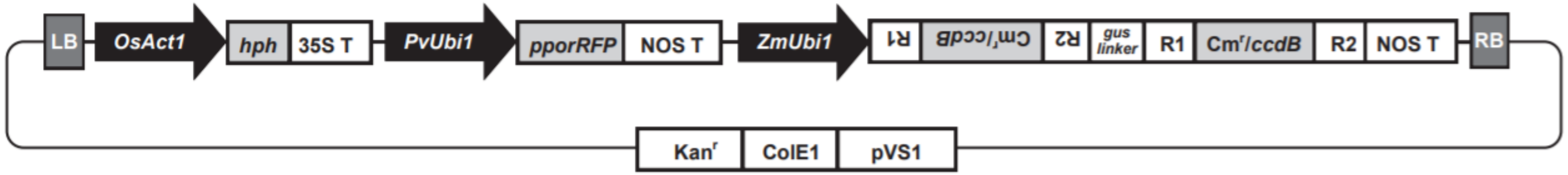

Mann et al., 2012

pANIC 8A

Figure S5. HCT1 and HCT2 transcripts in T0 transgenic plants in which HCT1 had been targeted by RNA interference. **a**, Single BdHCT1i lines. **b**, Double BdHCT1i:HCT2i lines. In panel a, all values shown for the HCT-RNAi lines are significantly different (P<0.05) from the wild-type control (WT) plants. In panel b, all values shown for the HCT-RNAi lines are significantly different (P<0.05) from the wild-type control (WT) plants except lines 13 and 19 for HCT1 and lines 2, 12 and 21 for HCT2.

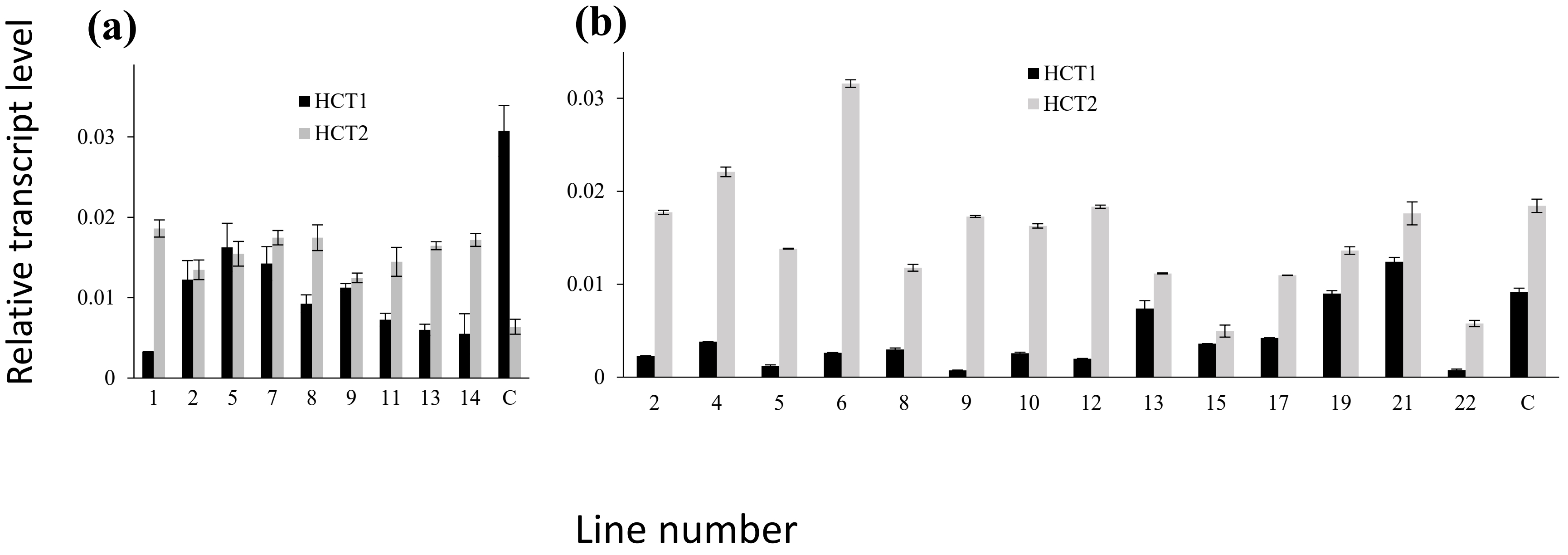

Figure S6. Lignin content and composition in T2 generation *B. distachyon* lines downregulated in HCT1 or HCT1 and HCT2. (**a**) Total lignin thisoacidolysis yields. (**b**) Percentages of hydroxyphenyl (H), guaiacyl (G) and syringyl (S) monomer units as determined by thioacidolysis. (**c**) H/T unit ratios in lignin. (**d**) S/G ratios in lignin.

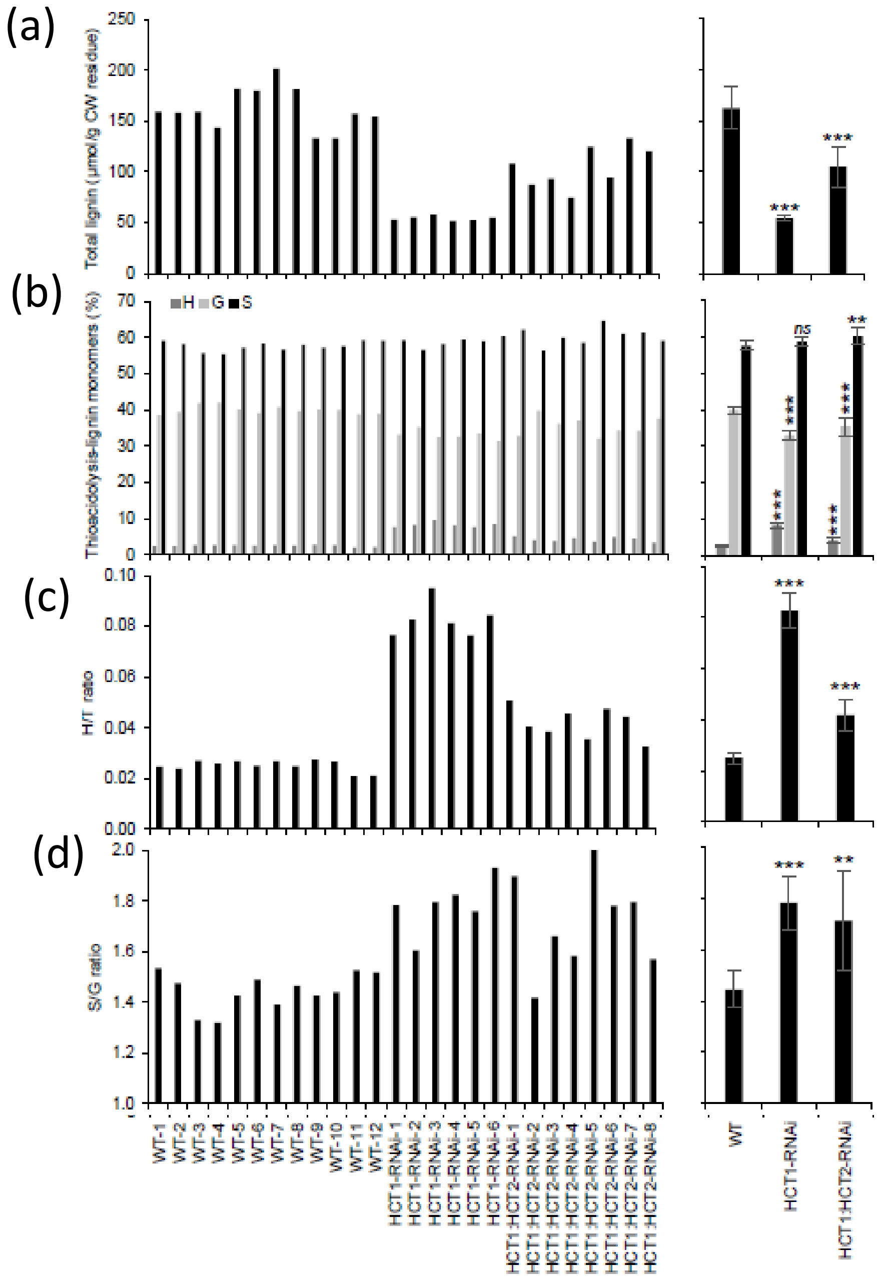

Figure S7. Gel permeation chromatography of acetylated lignins from wild-type (black), HCT1-RNAi (blue), and HCT1:HCT2 double RNAi (red) lines.

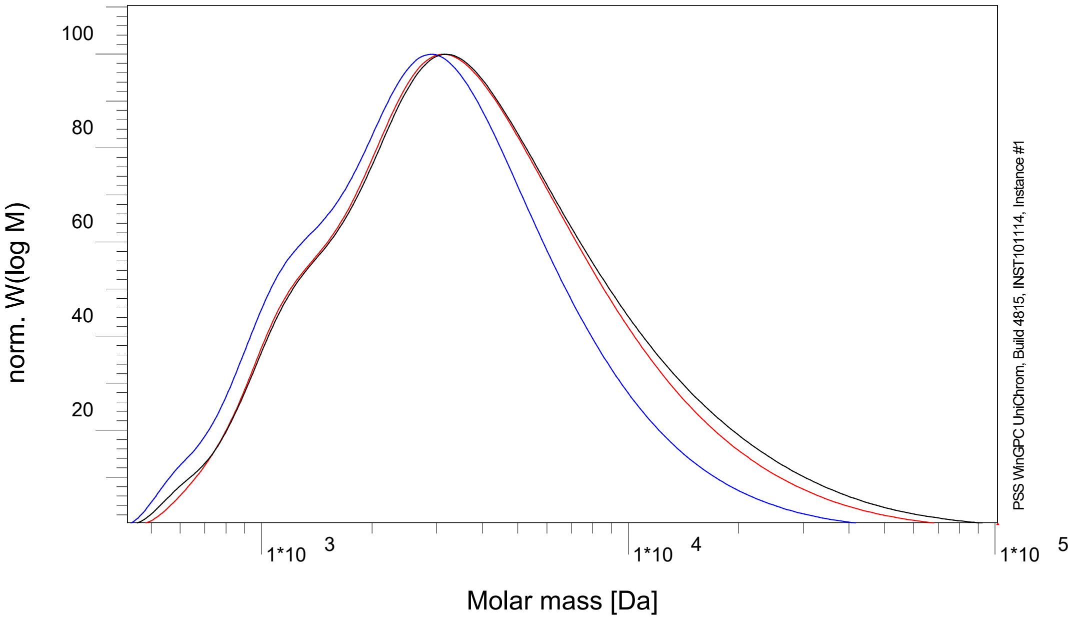

Table S1. Lignin content and composition of internodes 5 and 8 of *B. distachyon* stems harvested at 45 days after germination. Contents of syringyl (S), guaiacyl (G) and hydroxyphenyl (H) units were determined by thioacidolysis. T, total (S + G + H). Units are µmol/g cell wall residue. Bold, difference between internodes significant at $P < 0.05$.

**Lignin monomer**	**Internode 5**	**Internode 8**
**S**	**272.6** ± 1.5	247.3 ± 3.9
**G**	123.1 ± 4.8	116.1 ± 2.2
**H**	14.1 ± 0.9	13.3 ± 0.05
**S/G**	**2.2** ± 0.01	2.1 ± 0.00
**H/T**	0.04 ± 0.00	0.04 ± 0.00
**T**	**409.8** ± 4.1	376.7 ± 6.0

Table S2. Individual S:G and H: total lignin monomer ratios of both single and double *B. distachyon* HCT-RNAi lines from T0 and T1 generations. Data are derived from thioacidolysis yields in Fig. 3.

		S/G	H/T
**T0 singles**			
	HCT1i-1	2.37	0.055
	HCT1i-2	1.94	0.036
	HCT1i-5	2.47	0.052
	HCT1i-7	1.90	0.034
	HCT1i-8	2.34	0.057
	HCT1i-9	1.85	0.037
	Wild type	2.25	0.031
**T0 doubles**	HCT1i:HCT2i-5	1.37	0.073
	HCT1i:HCT2i-8	1.37	0.064
	HCT1i:HCT2i-22	1.27	0.070
	Wild type	1.39	0.032
**T1 singles**	HCT1i-1	1.02	0.068
	Wild type	0.97	0.043
**T1 doubles**	HCT1i:HCT2i-8	1.47	0.055
	Wild type	1.45	0.041

Table S3. Lignin composition and linkage types as determined by NMR analysis*.

	Control	HCT1i-1	HCT1i:HCT2i-8
S (%)	57.7	56.1	58.5
G (%)	38.8	33.1	30.9
H (%)	3.5	10.8	10.7
S/G ratio	1.5	1.7	1.9
FA	7.9	5.9	4.3
*p*CA	51.7	60.8	45.2
Tricin	10.5	13.0	10.6
β-O-4	52.2	45.3	49.8
β-5	1.36	1.21	1.59
β-β**	-	-	-
γ-acetylated β-β	8.45	8.97	9.81
Cinnamyl alcohol	1.48	1.52	0.84

*Data show mean values from three measurement (n = 3). Data were calculated based on contour signal integration divided by the integration of total S/G/H lignin units. ** under spectra noise level.

Table S4. Oligonucleotide primers used in the present work.

(**A**), primers for RNAi-targeted suppression;

(B), primers for RT-qPCR.

(**C**), primers for transgene integration analysis.

(D), primers for cDNA cloning and protein expression

	**Primer name**	**Primer sequence**	**Region length (bp)**
**A**	HCT1_RNAi-F<br>HCT1_RNAi-R	5'-TGGATTACCTGGAGCTGCA-3'<br>5'-TTCCGGAACTTCTCCATGTGC-3'	260
	HCT2_RNAi-F<br>HCT2_RNAi-R	5'-GACTCGGAGCCGCCGCAGGCCA-3'<br>5'-CGTGTTCGCGCCGTCCGCCAGCG-3'	360
**B**	HCT1_F<br>HCT1_R	5'-TCGCAGCCTTCCTCCTGAA-3'<br>5'-GGCTCCATCTTCTAACCCACTGG-3'	163
	HCT2_F<br>HCT2_R	5'-GAGAGCTCTTCGATCGATCGCA-3'<br>5'-GTAGCACCCGACCTCAACC-3'	184
	TUB_F<br>TUB_F	5'-GCCTTTGTCCACTGGTATGT-3'<br>5'-AACTCTGCACCAACCTCTTC-3'	110
**C**	Hyg_F<br>Hyg_R	5'-CGAAATTGCCGTCAACCAAGCTCT-3'<br>5'-CGACGTCTGTCGAGAAGTTT-3'	414
D	BdHCT1-F<br>BdHCT1-R	5’- CACCATGGCGATCACGGTGAAGC -3’<br>5’- TTACACCTCGAATATCAGCTTCCGGAAC -3’	1336
	BdHCT2-F<br>BdHCT2-R	5’- CACCATGAAGATCACGGTGCGGG -3’<br>5’- TCAGAAGTCGAAGATCATCTTCCGGAA -3’	1333
	AtHCT-F<br>AtHCT-R	5’- CACCATGAAAATTAACATCAGAGATTC -3’<br>5’- TCATATCTCAAACAAAAACTTCTC -3’	1306
	MtHCT1-F<br>MtHCT1-R	5’- CACCATGATCATAAACGTTAGAGATTC -3’<br>5’- TCAAATATCATACAAGAAGTCC -3’	1306
	MtHCT2-F<br>MtHCT2-R	5’- CACCATGTTGATCAACGTGAAAGAG -3’<br>5’- TCAAATGTCATAAAACAATTCC -3’	1303
